# Circulating IGF1 and IGF2 and SNP genotypes in men and pregnant and non-pregnant women

**DOI:** 10.1530/EC-14-0068

**Published:** 2014-08-28

**Authors:** K L Gatford, G K Heinemann, S D Thompson, J V Zhang, S Buckberry, J A Owens, G A Dekker, C T Roberts, on behalf of the SCOPE Consortium

**Affiliations:** School of Paediatrics and Reproductive Health, Robinson Research Institute, University of Adelaide Adelaide, South Australia, 5005 Australia

**Keywords:** human, IGF1, IGF2, pregnancy, SNP genotype

## Abstract

Circulating IGFs are important regulators of prenatal and postnatal growth, and of metabolism and pregnancy, and change with sex, age and pregnancy. Single-nucleotide polymorphisms (SNPs) in genes coding for these hormones associate with circulating abundance of IGF1 and IGF2 in non-pregnant adults and children, but whether this occurs in pregnancy is unknown. We therefore investigated associations of plasma IGF1 and IGF2 with age and genotype at candidate SNPs previously associated with circulating IGF1, IGF2 or methylation of the *INS* *–* *IGF2* *–* *H19* locus in men (*n*=134), non-pregnant women (*n*=74) and women at 15 weeks of gestation (*n*=98). Plasma IGF1 concentrations decreased with age (*P*<0.001) and plasma IGF1 and IGF2 concentrations were lower in pregnant women than in non-pregnant women or men (each *P*<0.001). SNP genotypes in the *INS* *–* *IGF2* *–* *H19* locus were associated with plasma IGF1 (*IGF2* rs680, *IGF2* rs1004446 and *IGF2* rs3741204) and IGF2 (*IGF2* rs1004446, *IGF2* rs3741204 and *H19* rs217727). In single SNP models, effects of *IGF2* rs680 were similar between groups, with higher plasma IGF1 concentrations in individuals with the GG genotype when compared with GA (*P*=0.016), or combined GA and AA genotypes (*P*=0.003). SNPs in the *IGF2* gene associated with IGF1 or IGF2 were in linkage disequilibrium, hence these associations could reflect other genotype variations within this region or be due to changes in *INS* *–* *IGF2* *–* *H19* methylation previously associated with some of these variants. As IGF1 in early pregnancy promotes placental differentiation and function, lower IGF1 concentrations in pregnant women carrying *IGF2* rs680 A alleles may affect placental development and/or risk of pregnancy complications.

## Introduction

The insulin-like growth factors (IGFs), IGF1 and IGF2, are important regulators of placental and foetal development, as well as postnatal growth and metabolism. In humans, circulating IGF1 peaks in adolescence and then falls with age, whereas IGF2 concentrations remain fairly stable after puberty (1). The pubertal peak in plasma IGF1 occurs 1–2 years earlier in girls than in boys, resulting in higher circulating IGF1 concentrations in girls than in boys through adolescence (2, 3). Plasma IGF1 concentrations are fairly similar in men and women (2, 4) but slightly lower circulating IGF1 concentration in adult women than in men has been reported in large studies (3, 5). Plasma IGF2 is similar in adolescent and young adult men and women (6, 7), but whether IGF2 remains similar between sexes throughout ageing is unknown.

IGF abundance is also altered by pregnancy. Variable changes in circulating IGF1 during the first two trimesters of human pregnancy have been reported, with modest increases of 25–40% compared with non-pregnant women (8) or a gradual overall rise with increasing gestation and highly variable concentrations between women in cross-sectional studies (9, 10). Longitudinal studies have shown stable concentrations from early pregnancy (8–10 weeks) until after 30 weeks of gestation (11, 12), or decreased concentrations in the first trimester up until 24 weeks of gestation compared with pre-conception (13, 14, 15). All these studies agree that maternal circulating IGF1 is 45–200% higher in the third trimester when compared with non-pregnant women, early pregnancy or pre-conception (8, 9, 10, 11, 12, 13, 14, 15, 16). Fewer studies have characterised circulating IGF2 abundance throughout pregnancy. Gargosky *et al*. (8) reported much higher plasma IGF2 concentrations than IGF1 concentrations in pooled plasma from pregnant women, measured by RIA after HPLC separation of samples to completely remove IGF-binding proteins. IGF2 concentrations were highly variable between different stages of pregnancy, but as these were analysed in pooled samples it is difficult to draw conclusions about changes across pregnancy (8). In an early cross-sectional study, plasma IGF2 concentrations were higher in women in the third trimester when compared with the first trimester of pregnancy and decreased *post partum* (10). Two longitudinal studies each measuring IGF2 by RIA after acid–ethanol extraction reported decreases of ∼10% in plasma IGF2 in the first trimester compared with concentrations in the same women before pregnancy (14, 15). As pregnancy progressed, plasma IGF2 returned to pre-conception concentrations (14) or increased to ∼10% above pre-conception concentrations (15). In addition to effects on maternal metabolism, IGFs act as endocrine signals to enhance placental function and foetal growth (reviewed by (17)). We have previously directly demonstrated the endocrine actions of maternal IGFs to enhance placental differentiation and function and hence foetal growth in the guinea pig (18, 19, 20). Consistent with this, late pregnancy maternal circulating IGF1 concentration is reduced in human pregnancies complicated by IUGR compared with those with normally grown neonates (11, 21).

Genetic variation also impacts the IGF axis and circulating IGF1 and IGF2 differ between individuals according to their genotype at single-nucleotide polymorphisms (SNPs) in the genes for *IGF1*, *IGF2* and the IGF1 receptor (*IGF1R*). Within the *IGF1* locus, rs12579108 is weakly associated with plasma IGF1 in children in combination with other SNPs (22), while the rare C allele of the *IGF1* rs7965399 SNP was associated with increased plasma IGF1 concentrations in older women but not with plasma IGF1 concentrations in other populations (23, 24, 25). Consistent with a positive effect of the *IGF1* rs7965399 C allele on IGF1, this allele was also associated with a trend towards higher IGF1 concentration in breast tumours (26). Circulating IGF1 is also associated with genotype at the *IGF1R* rs2229765 SNP, which is predicted to regulate alternative splicing of *IGF1R* (27). The AA genotype at this SNP predicts lower plasma IGF1 concentrations in adult men and women compared with GG individuals in most (28, 29, 30), but not all, studies (31, 32), with lower plasma IGF1 concentrations also reported in AG heterozygotes (28). The AA genotype also predicts increased longevity (28, 29, 30), and shorter male adult height (33), consistent with decreased IGF1 action in these individuals, as absence of IGF1 signalling through IGF1R reduces postnatal growth (34), and IGF1 deficiency predicts longevity (35).

**Figure 1:**
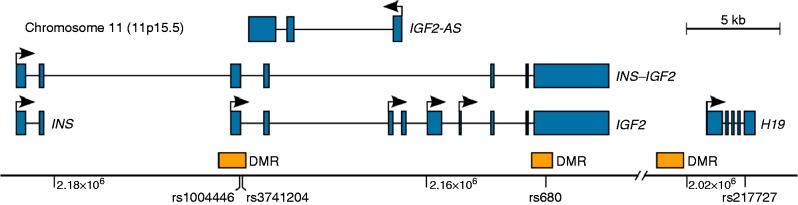
Schematic of the *Homo sapiens* *INS* *–* *IGF2* *–* *H19* locus. Exons are represented as blue boxes with intronic regions between exons as black lines. Black arrows above exons show transcription start sites and direction of transcription. Orange boxes indicate the approximate location of differentially methylated regions (DMRs). The *x*-axis shows genomic position in base pairs for human chromosome 11 and the position of single-nucleotide polymorphisms (SNPs, denoted by rs number) investigated in this study. This representation is based on human reference genome hg19, dbSNP 138 and RefSeq transcripts.

*IGF2* is located in an imprinted gene cluster on chromosome 11p15.5 (Fig. 1), containing genes for *H19*, *IGF2*, insulin (*INS*), tyrosine hydroxylase (*TH*) and an antisense *IGF2* gene overlapping with *IGF2* (*IGF2-AS*). The *H19* long non-coding RNA in this cluster is maternally expressed and this imprinting appears to remain stable with age (36, 37). *IGF2* and *H19* are reciprocally imprinted during early development and, in foetal, placental and many adult tissues, *IGF2* is paternally expressed from the P0, P2, P3 and P4 promoters (36, 37, 38). P1 promoter transcripts of *IGF2* are expressed from both parental alleles and IGF2 is expressed bi-allelically in liver from older infants and adults, where imprinting of *IGF2* is not closely co-regulated with that of *H19* (36, 37). We have recently reported discordant imprinting of *IGF2* and *H19* in first trimester human placenta at 6 weeks of gestation, where expression of *IGF2* is mono-allelic but imprinting of *H19* is highly variable (39). Individuals with Beckwith–Wiedemann syndrome and loss of imprinting at this locus, who therefore express maternal and paternal *IGF2* alleles, often have pre- and postnatal overgrowth, suggesting increased IGF2 availability (reviewed by (40)). This suggests that SNPs associated with altered DNA methylation at this locus may also regulate circulating IGF2. Indeed, plasma IGF2 concentrations have previously been associated with genotype at two SNPs associated with *INS* *–* *IGF2* *–* *H19* methylation. Specifically, *IGF2* rs680 and *H19* rs217727 SNPs strongly correlate with methylation of multiple CpG sites within the *IGF2* and *H19* differentially methylated regions respectively (41). Circulating IGF2 concentrations were higher in individuals homozygous for the A allele at *IGF2* rs680 (ApaI), compared with those homozygous for the G allele in middle-aged men (42). The A allele is also part of a haplotype of four SNPs that are positively associated with IGF2 protein content of placentas collected at term (43). Others found no effect of *IGF2* rs680 on plasma IGF2 concentrations in studies of middle-aged to elderly men and women (44, 45, 46). Conversely, the *IGF2* rs680 G allele was associated with higher *IGF2* mRNA expression in leukocytes (47). Neonatal *IGF2* rs680 A alleles were associated with lower birth weight than G alleles in Brazilian and Japanese populations (48, 49). By contrast, maternal (50) or neonatal (43, 50, 51) *IGF2* rs680 genotypes were not associated with birth weight in Caucasian populations. A paternally inherited foetal A allele at *IGF2* rs680 was, however, associated with higher maternal circulating glucose post-challenge at 27–29 weeks of gestation (43), consistent with an effect of this allele on maternal adaptation to pregnancy. Only one study has investigated differences in circulating IGF2 with the *H19* rs217727 SNP. The presence of one or more T alleles at *H19* rs217727 in women was positively associated with birth size and cord blood IGF2 in their neonates, with the TT genotype relatively rare (<5%) in mothers and newborns (52). Methylation of the *INS* *–* *IGF2* *–* *H19* locus also differs according to genotype at *IGF2* rs1004446 (41) and *IGF2* rs3741204. *IGF2* rs3741204 is located within the P3 promoter of *IGF2* within the DMR0 region that affects imprinting of *IGF2* and *H19*. The A allele is observed in two different four SNP haplotypes associated with either increased or decreased methylation of the *INS* *–* *IGF2* *–* *H19* locus in Beckwith–Wiedemann syndrome (53). Associations of *IGF2* rs1004446 and *IGF2* rs3741204 with circulating IGF2 have not been reported as yet.

Although relationships between SNP genotype and circulating IGFs have been previously investigated in non-pregnant subjects, no studies to date have reported their associations in pregnant women, when circulating IGF concentrations regulate placental and foetal growth and development (17). We therefore investigated whether relationships between circulating IGF1 and IGF2 abundance and SNP genotypes previously associated with circulating IGFs (*IGF1* rs12579108, *IGF1* rs7965399, *IGF1R* rs2229765, *IGF2* rs680 and *H19* rs217727) and/or methylation of the *INS* *–* *IGF2* *–* *H19* locus (*IGF2* rs680, *IGF2* rs1004446, *IGF2* rs3741204 and *H19* rs217727) differ among men, pregnant women and non-pregnant women.

## Materials and methods

### Study populations and sample collection

Circulating IGFs and genotype data from Caucasian subjects within two independent studies are included in the present analysis. Non-pregnant women were from a general population cohort and pregnant women from a subset of the Adelaide Screening for Pregnancy Endpoints (SCOPE) cohort, who had a normal pregnancy outcome, as described below, while male subjects were from the general population or partners of the pregnant women (Table 1).

Healthy, non-pregnant adults were recruited from the general population in Adelaide, South Australia, and gave informed consent for participation in the study. Inclusion criteria were age (18–60 years) and not taking regular medication other than the oral contraceptive pill. First-degree (siblings, parent–child) and second-degree relatives (cousins) were excluded. Ethics approval for this work was given by the University of Adelaide Human Research Ethics Committee (H-021-2005).

Pregnant women and their partners were recruited from a nested case–control study within the Adelaide SCOPE cohort, an international prospective cohort study recruiting patients in Australia, New Zealand (ACTRN12607000551493, Australian and New Zealand Clinical Trials Registry), UK and Ireland, which aims to predict and prevent the major complications of late pregnancy (54). Women who were nulliparous with a singleton pregnancy at <15 weeks of completed gestation and with no more than two previous terminations of pregnancy or miscarriages were recruited into the Adelaide cohort after providing written informed consent at the Lyell McEwin Hospital antenatal clinic (Elizabeth Vale, SA, Australia). This study includes only women who had an uncomplicated pregnancy, defined as women who remained normotensive (<140 mmHg systolic and/or <90 mmHg diastolic prior to labour), showed no proteinuria, delivered a live born baby who was not small for gestational age after 37 weeks of completed gestation and had no other signs of pregnancy complications. The pregnant women in this study were the 98 women in whom genotype and circulating IGF data were available, from a cohort of 133 control women with normal pregnancy outcomes, BMI-matched to pregnant women who later developed preeclampsia or gestational hypertension (55) or gestational diabetes or who delivered before 37 weeks of completed gestation (preterm) or a small-for-gestational-age infant. Ethics approval for this work was given by the Ethics of Human Research Committee, Central Northern Adelaide Health Service (REC 1712/5/2008).

Non-fasting blood samples were collected by venepuncture from women at 15 weeks of gestation, and their partners sometimes, during the women's pregnancy, and from general population subjects. Samples were collected into EDTA tubes and placed on ice, before centrifugation at 2400 ***g*** for 10 min at 4 °C. Plasma and buffy coats were harvested and stored at −80 °C for subsequent analyses.

### Plasma IGF1 and IGF2 analyses

Concentrations of plasma IGF1 and IGF2 were measured by RIA after separation of IGFs and IGFBPs by size-exclusion HPLC under acidic conditions (8, 56). Four fractions of eluate (fraction 1, containing IGFBPs; fraction 2, inter-peak; fraction 3, containing IGFs and fraction 4, post-peak) were routinely collected for each acidified plasma sample, using collection times based on elution times of ^125^I-IGF1 and IGF immunoreactivity. Recovery of ^125^I-IGF1 was 88.0±1.1% for five HPLC runs of human plasma. Samples were assayed in triplicate. Plasma IGF1 concentrations were measured by analysis of neutralised HPLC fraction 3, in an RIA specific for IGF1, using a rabbit polyclonal antibody to human IGF1 (GroPep, Adelaide, SA, Australia). Plasma IGF2 concentrations were measured by analysis of HPLC fraction 3 in an RIA specific for IGF2 (57), using a mouse MAB against rat IGF2, which has 100% cross-reactivity with human IGF2 and <10% cross-reactivity with human IGF1 (anti-IGF2 clone, Millipore, St Charles, MO, USA). Inter- and intra-assay coefficient of variation (CV) values for HPLC separation and IGF1 RIA of a non-pregnant female QC human plasma pool were <19 and <14% respectively (14 assays). Inter- and intra-assay CV values for HPLC separation and IGF2 assays were <15 and <10% respectively (13 assays).

### DNA extraction and genotyping

A series of SNPs previously shown to affect circulating abundance of IGF1 (*IGF1* rs1257918, *IGF1* rs7965399 and *IGF1R* rs2229765), circulating abundance of IGF2 (*IGF2* rs680 and *H19* rs217727), and/or methylation of the *INS* *–* *IGF2* *–* *H19* locus (*IGF2* rs680, *IGF2* rs3741204, *IGF2-AS* rs1004446 and *H19* rs217727) were genotyped in extracted DNA. DNA was extracted from buffy coats using the X-Tractor Gene (Corbett Robotics Pty Ltd, Eight Mile Plains, QLD, Australia) following the manufacturer's instructions or by the Australian Genome Research Facility (AGRF, Adelaide, SA, Australia) using the Machery Nagel Nucleospin 96-well format. Genotyping was performed at AGRF (Brisbane, OLD, Australia) using the Sequenom MassARRAY System. The assay used the iPLEX Gold homogenous MassExtend (hME – single base extension) reaction. Oligonucleotides obtained were used to process samples in the multiplex format, then printed onto Spectro CHIPs and analysed by MALDI–TOF mass spectrometry. All genotypes were in Hardy–Weinberg equilibrium and the genotype pass rate was >96% across all SNPs.

### Statistical analysis

Statistical analyses were performed using IBM SPSS Statistics v 21. Circulating IGF concentrations were log transformed before analyses to overcome unequal variances. Effects of group (male, non-pregnant female or pregnant female) on circulating IGF concentrations were analysed by ANOVA, including age as a covariate, and groups compared using the Bonferroni's correction for multiple comparisons. In initial analyses, BMI did not alter circulating IGF concentrations when included as a covariate in univariate analyses for effects of group or when included in preliminary regression analyses (data not shown) and BMI was therefore not included as a covariate in final analyses. Effects of group on SNP frequencies were assessed by the *χ* ^2^ analysis, or by Fisher's exact test for rare alleles. Predictors of plasma IGF concentrations were derived by stepwise backward linear regression commencing from a model including group, age, and common allele frequency for each SNP. Age was included as a covariate in models with circulating IGF1 as an outcome. For each SNP identified as significant or approaching significance (*P*<0.1) in stepwise linear regressions, we tested the effects of SNP genotype, group and interactions on circulating IGF concentrations in two-way ANOVA and performed pair-wise cross-tabulation to determine whether these SNPs were in linkage disequilibrium.

## Results

### Circulating IGF1 and IGF2

**Figure 2:**
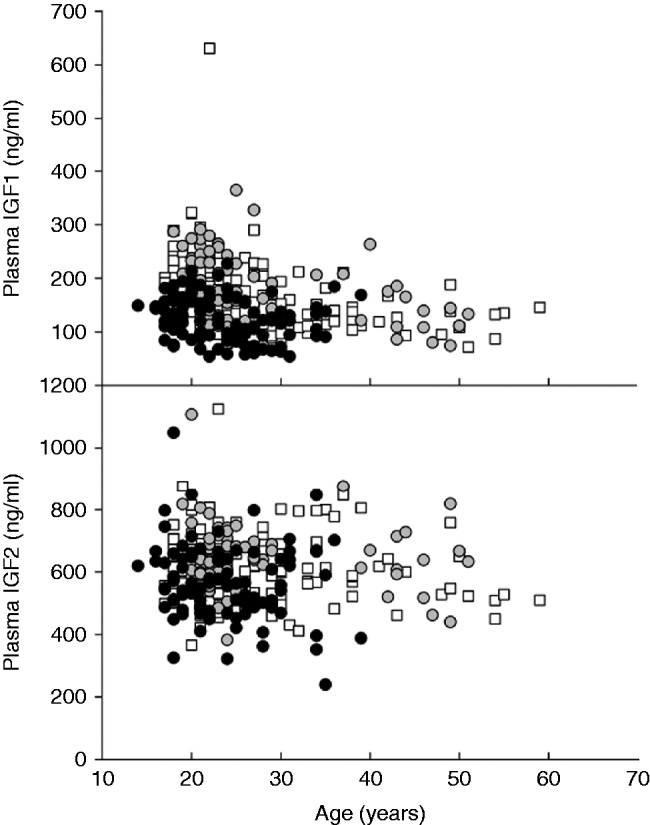
Circulating plasma IGF1 and IGF2 in men (white squares), non-pregnant women (grey circles) and at 15 weeks of gestation in pregnant women (black circles).

Plasma IGF1 concentrations (Fig. 2A) decreased with age (*P*<0.001) and differed between groups (*P*<0.001). Plasma IGF1 concentrations in women at 15 weeks of gestation were 31 and 45% lower than those in men or non-pregnant women respectively (*P*<0.001 for both). Plasma IGF2 concentrations (Fig. 2B) tended to decrease with age (*P*=0.078) and differed between groups (*P*<0.001). Plasma IGF2 concentrations in women at 15 weeks of gestation were 9 and 12% lower than those in men or non-pregnant women respectively (*P*<0.001 for both). Neither plasma IGF1 nor IGF2 concentrations differed between men and non-pregnant women. Effects of age on plasma IGF1 and IGF2 concentrations were similar between groups.

### SNP genotype frequencies

Frequencies of individuals homozygous for the rare allele of the seven SNPs investigated varied from 18% for *IGF1R* rs2229765 to 0% for *IGF1* rs12579108 and *IGF1* rs7965399 (Table 2). Genotype frequencies did not differ among men, non-pregnant women and pregnant women (Table 2).

### Effects of SNP genotype on circulating IGF1 concentrations

In overall regression models including data from all subjects, plasma IGF1 differed between groups (*P*<0.001), decreased with age and differed with a common allele frequency of three SNPs in the *INS* *–* *IGF2* *–* *H19* gene locus (Table 3). Overall, plasma IGF1 correlated positively with the numbers of the common G allele of *IGF2* rs680 and the common C allele of *IGF2* rs1004446, and correlated negatively with the numbers of the common A allele of *IGF2* rs3741204. Similar correlations of plasma IGF1 with age and SNP frequencies were observed in non-pregnant women (Table 3). Within men alone, plasma IGF1 correlated negatively with age and was not correlated with the allele number for any SNP (Table 2). In pregnant women, plasma IGF1 correlated negatively with age and positively with the number of the common G allele of *IGF2* rs680 (Table 3).

**Figure 3:**
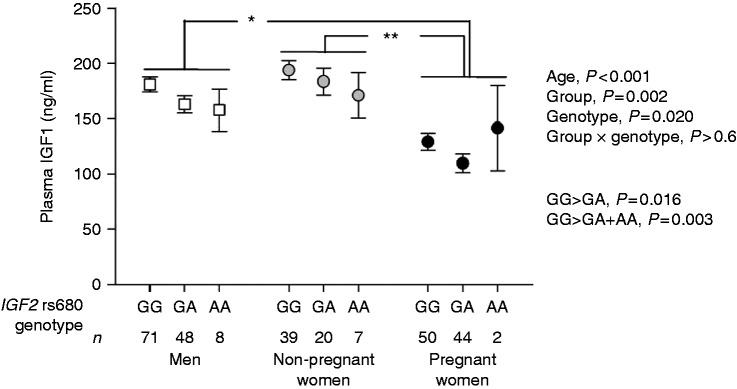
Plasma IGF1 according to *IGF2* rs680 SNP genotype in men (white squares), non-pregnant women (grey circles) and at 15 weeks of gestation in pregnant women (black circles). Plasma IGF1 data are estimated means and s.e.m. adjusted to an average age of 26.2 years. Differences between groups are indicated as follows: *P< 0.05; **P<0.01.

In separate analyses of associations of each SNP (*IGF2* rs680, *IGF2* rs1004446 and *IGF2* rs3741204), plasma IGF1 differed between groups (*P*≤0.002 for each model) and correlated negatively with subject age (*P*<0.001 for each model). Plasma IGF1 concentration differed between *IGF2* rs680 genotypes, being higher in GG individuals compared with GA individuals alone (*P*=0.016) or with GA and AA genotypes combined (*P*=0.003, Fig. 3). Effects of *IGF2* rs680 genotype on plasma IGF1 concentration did not differ between groups. Plasma IGF1 did not differ between *IGF2* rs1004446 and *IGF2* rs3741204 genotypes.

### Effects of SNP genotype on circulating IGF2 concentrations

Overall, plasma IGF2 concentrations differed between groups (*P*=0.002) and with common allele numbers of three SNPs in the *INS* *–* *IGF2* *–* *H19* gene locus (Table 3), but were not affected by age. Plasma IGF2 correlated positively with the number of the common C allele of *IGF2* rs1004446 and negatively with the numbers of the common A allele of *IGF2* rs3741204 and the common C allele of *H19* rs217727 (Table 3). Within men alone, non-pregnant women alone, or pregnant women alone, plasma IGF2 was not correlated with allele frequencies for any SNP (Table 3).

In separate analyses of associations of each SNP with plasma IGF2, plasma IGF2 differed between groups (*P*≤0.002 for each model), but did not differ among *IGF2* rs3741204, *IGF2* rs1004446 or *IGF2* rs3741204 genotypes.

### Linkage analysis

The three SNPs identified in stepwise backward regression as predictive of circulating IGF1 were in linkage disequilibrium, particularly strong between *IGF2* rs3721204 and *IGF2* rs1004446. Within the overall population, 97.8% of individuals (*P*<0.001) with AA, AG and GG genotypes at *IGF2* rs3721204 had CC, CT and TT genotypes, respectively, at *IGF2* rs1004446, located 235 nucleotides distant within the *IGF2* gene (Fig. 1). Genotype of *IGF2* rs680 shared 34.4% concordance with *IGF2* rs1004446 (*P*=0.007) and 32.0% concordance with *IGF2* rs3721204 (*P*=0.016). Two of the three SNPs identified in stepwise backward regression as predictive of circulating IGF2 were in linkage disequilibrium, *IGF2* rs3721204 and *IGF2* rs1004446, as described above. Genotype at *H19* rs217727 tended towards concordance with *IGF2* rs1004446 genotype (*P*=0.053) but not with *IGF2* rs3721204 genotype.

## Discussion

This study provides the first comparison of circulating IGF abundance in men, non-pregnant and pregnant women within the same population. Similar plasma IGF1 concentrations in non-pregnant women and men and falling plasma IGF1 concentrations with age were consistent with previous information, while a lack of change in plasma IGF2 in these mature adults with sex or age extends previous findings of similar plasma IGF2 abundance in male and female children and adolescent humans. IGF1 and IGF2 concentrations in circulation were both lower in pregnant women at 15 weeks of gestation than in either men or non-pregnant women. For the first time, we identified differences in circulating IGF1 between individuals according to common allele numbers in three linked SNPs in the *INS* *–* *IGF2* *–* *H19* locus. Associations between circulating IGF1 and *IGF2* rs680 genotype remained significant in single SNP models and were consistent among men, non-pregnant women and pregnant women. This suggests that effects of SNP genotype in the *INS* *–* *IGF2* *–* *H19* locus are consistent between sexes and unaffected by pregnancy. Overall, plasma IGF2 concentrations were also predicted by common allele numbers of three SNPs in the *INS* *–* *IGF2* *–* *H19* locus, including two SNPs for which the common allele number also correlated with plasma IGF1. Our results show genotypes in the *IGF2* region of the *INS* *–* *IGF2* *–* *H19* locus associated with circulating IGF1 and IGF2 concentrations, which requires confirmation in additional independent populations. This is the first report of lower circulating IGF1 concentration in pregnant women with the A allele at *IGF2* rs680 SNP genotype. Given the endocrine actions of maternal IGFs in pregnancy, we hypothesise that *IGF2* rs680 genotype may affect placental development and function and maternal adaptation to pregnancy. We are currently exploring these effects in women who experienced pregnancy complications in a separate study.

Circulating IGF1 concentrations were lower in women at 15 weeks of gestation than in either men or non-pregnant women in this study. Our data, obtained using a methodology that completely separates IGFs from IGFBPs before assay and prevents IGFBP interference in IGF assays, are consistent with previous reports of reductions in circulating IGF1 during early–mid pregnancy from longitudinal studies (14, 15). We hypothesise that this decrease of ∼45% in circulating IGF1 at 15 weeks of gestation, compared with non-pregnant women, largely reflects increased negative feedback on IGF1 production, due to increased IGF1 bioavailability despite reduced total IGF1 concentrations. Proteolysis of IGFBP3 and other IGFBPs increases rapidly in human pregnancy by ∼6–8 weeks of gestation and decreases their binding affinity for IGFs, which increases circulating concentrations of free or unbound IGFs available to bind receptors (58, 59, 60). The placenta produces two metalloproteinases which proteolyse IGFBPs: pregnancy-associated plasma protein A (PAPPA), which cleaves IGFBP4 and to a lesser extent IGFBP5 (reviewed by (61)), and PAPPA2, which mostly cleaves IGFBP5 (62). Haemodilution, due to expansion of maternal blood volume in early pregnancy, may also account for ∼20–25% of the fall in circulating IGF1 concentration that we observed (14).

The increases in circulating IGF1 concentration reported in later pregnancy (8, 9, 11, 14, 16) are probably a response to increasing maternal circulating GH concentrations stimulated by rapid increases in placental GH production during the second trimester (63). These result in elevated, non-pulsatile GH concentration in maternal circulation from 17–24 weeks of gestation (63, 64). Plasma IGF1 and IGF2 normalise across gestation in women who are deficient in pituitary GH (65), implying that placental GH is a major regulator of IGF abundance during pregnancy. Furthermore, the human placenta itself expresses *IGF1* and *IGF2*, and *IGF1* gene and protein expression occurs on both maternal and foetal sides of the human placenta (66, 67), and placental tissues might therefore be a source of circulating IGFs during pregnancy. This study is the first to show that IGF1 falls with age in pregnant women, while the decrease with age in non-pregnant women is consistent with previous reports that IGF1 falls from young to old adulthood (4). Plasma IGF1 did not differ between non-pregnant women and men, consistent with most previous studies, where although the pattern of change in circulating IGF1 throughout puberty differed between sexes, plasma concentrations are similar in men and women as young and old adults (2, 4). Small sex differences were evident in a recent multi-centre study with over 15 000 subjects, where circulating IGF1 concentrations were slightly lower in women than men (5).

The 12% lower IGF2 concentration in pregnant women at 15 weeks of gestation compared with non-pregnant women at similar ages is consistent with the magnitude of reductions in circulating IGF2 at similar stages of pregnancy reported previously in longitudinal studies (14, 15). This early-pregnancy fall in IGF2 was explained by haemodilution (14) due to expansion of maternal blood volume in early pregnancy. Our findings across the adult age range in this study extend those from studies in children throughout puberty and up to young adulthood (6, 7), where plasma IGF2 concentrations also do not change with age or differ between sexes.

Our results provide the first evidence that SNP genotypes in the *INS* *–* *IGF2* *–* *H19* locus associate with circulating concentrations of IGF1, as well as IGF2. The number of *IGF2* rs680 common G alleles was positively associated with circulating IGF1 concentrations overall and in non-pregnant and pregnant women analysed separately. Associations of genotypes at this SNP with circulating IGF1 were robust and did not differ among men, non-pregnant or pregnant women in univariate analysis. In this study, individuals with the *IGF2* rs680 GA or GA+AA genotypes consistently had lower plasma IGF1 concentrations than those homozygous for the G allele. The G allele has previously been associated with lower circulating IGF2 concentrations than the A allele in middle-aged men (42), but we did not find any association between genotype at this SNP and plasma IGF2 in this study. This suggests that associations between *IGF2* rs680 and circulating IGF1 do not reflect competition with circulating IGF2 for IGFBP-binding sites and consequent effects on circulating half-life. *IGF2* is imprinted and only the paternally inherited allele is expressed in many, but not all, tissues postnatally (37). Differences in circulating IGF1 between GG and GA+AA genotypes observed in this study are therefore likely to be smaller than the actual effects of the paternally expressed alleles of *IGF2* rs680, as the GA heterozygotes will include individuals with paternally inherited A and G alleles. As these three SNPs in *IGF2* were in linkage disequilibrium within this population, associations of circulating IGF1 with *IGF2* rs680 SNP genotype could reflect variation anywhere within this region. Nevertheless, they do suggest that genotypes at this locus might affect placental development and maternal adaptation to pregnancy via effects on IGF1 or IGF2 abundance, given that both these peptides are endocrine regulators of placental growth and differentiation (17). Further studies are needed to confirm these effects of *INS* *–* *IGF2* *–* *H19* locus SNP genotypes on circulating IGF1, to investigate underlying mechanisms and assess potential effects on the placenta and mother.

Across all groups combined (*n*=307), SNP genotype at *IGF2* rs3741204, *H19* rs217727 and *IGF2* rs1004446 correlated with circulating plasma IGF2 in multiple linear regression analyses. A negative association of the common C allele of *H19* rs217727 with circulating IGF2 concentrations is consistent with the reported effects of this SNP on cord blood IGF2 (52). This study provides the first evidence that SNP genotype at *IGF2* rs3741204 or *IGF2* rs1004446 may affect circulating IGF2. Genotypes at these two SNPs were extremely tightly linked in this population, consistent with their proximity within the *IGF2* and *IGF2-AS* genes at 235 nucleotides apart. These associations might therefore reflect effects of either of these SNPs or of other SNPs in this linkage region. Our findings, together with previously reported associations between *IGF2* rs680 genotype and circulating IGF2 in one study of middle-aged men (42), are also consistent with the hypothesis that SNPs that are associated with altered methylation of the *INS* *–* *IGF2* *–* *H19* locus, such as *IGF2* rs3741204, *IGF2* rs1004446 and *IGF2* rs680 (41, 53), may affect *IGF2* expression and secretion. Further investigations are required to identify which SNP or SNPs in this region alter(s) the methylation and expression of *IGF2*. The loss of associations of any SNPs with circulating IGF2 in men (*n*=134), non-pregnant women (*n*=74) or pregnant women (*n*=98) in regression models run separately in each group, or when analysing effects of genotype and group separately for each SNP, probably reflects the limited power due to smaller sample sizes within each sub-group of this study. Comparing effects of these three SNPs between sexes and in pregnant and non-pregnant populations will require additional, larger studies.

In conclusion, plasma IGF1 and IGF2 concentrations were lower in pregnant women at 15 weeks of gestation than in men or non-pregnant women and did not differ between adult men and non-pregnant women. We have identified SNPs in the *INS* *–* *IGF2* *–* *H19* locus associated with circulating IGF1, as well as IGF2. Associations between *IGF2* rs680 and circulating IGF1 did not differ among men, non-pregnant and pregnant women. As maternal circulating IGFs in early–mid pregnancy are endocrine regulators of placental development and function, these genotypes may also predict foetal growth and risk for pregnancy complications. Further studies are needed to confirm these putative effects of SNPs in the *INS* *–* *IGF2* *–* *H19* locus on circulating IGF1 and IGF2 concentrations and identify the underlying mechanisms.

## Author contribution statement

K L Gatford, J A Owens, C T Roberts and G A Dekker conceived and designed the research project. K L Gatford, G K Heinemann, S D Thompson and J V Zhang performed sample and data analysis. K L Gatford and C T Roberts drafted the manuscript. All authors contributed to critical revision and approved the final draft of the manuscript.

## Figures and Tables

**Table 1:** Subject characteristics. This study includes only Caucasian individuals with data for circulating IGFs and genotype. Subject characteristics are expressed as median (range).

	**Men**	**Non-pregnant women**	**Pregnant women**
Number	134	74	98
Age (years)	25.0 (17–59)	23.5 (18–51)	23.0 (14–39)
Body weight (kg)	82.0 (55.0–133.1)	64.0 (43.0–100.0)	72.5 (44.8–125.1)
Height (m)	1.81 (1.64–1.96)	1.66 (1.53–1.78)	1.65 (1.49–1.82)
BMI (kg/m^2^)	24.7 (18.0–37.0)	23.1 (17.7–39.5)	26.8 (17.7–44.8)

**Table 2:** SNP genotype frequencies.

**SNP and population**	**Genotype**, *n* (%)			Significance*
*IGF1* rs12579108	CC	CA	AA	0.383
Men	130 (98)	2 (2)	0 (0)	
Non-pregnant women	73 (99)	1 (1)	0 (0)	
Pregnant women	93 (96)	4 (4)	0 (0)	
Total	296 (98)	7 (2)	0 (0)	
*IGF1* rs7965399	TT	TC	CC	0.591
Men	124 (95)	6 (5)	0 (0)	
Non-pregnant women	68 (92)	6 (8)	0 (0)	
Pregnant women	86 (93)	6 (7)	0 (0)	
Total	278 (94)	18 (6)	0 (0)	
*IGF2* rs680	GG	GA	AA	0.101
Men	71 (56)	48 (38)	8 (6)	
Non-pregnant women	39 (59)	20 (30)	7 (11)	
Pregnant women	50 (52)	44 (46)	2 (2)	
Total	160 (55)	112 (39)	17 (6)	
*IGF2* rs3741204	AA	AG	GG	0.273
Men	54 (43)	60 (47)	13 (10)	
Non-pregnant women	17 (29)	34 (58)	8 (14)	
Pregnant women	34 (37)	43 (46)	16 (17)	
Total	105 (38)	137 (49)	37 (14)	
*IGF2* rs1004446	CC	CT	TT	0.698
Men	58 (45)	58 (45)	14 (11)	
Non-pregnant women	30 (42)	32 (45)	9 (13)	
Pregnant women	36 (37)	45 (46)	16 (16)	
Total	124 (42)	135 (45)	39 (13)	
*H19* rs217727	CC	CT	TT	0.756
Men	83 (63)	45 (34)	3 (2)	
Non-pregnant women	45 (62)	24 (33)	4 (5)	
Pregnant women	62 (65)	29 (31)	4 (4)	
Total	190 (64)	98 (33)	11 (4)	
*IGF1R* rs2229765	GG	GA	AA	0.222
Men	44 (34)	65 (50)	22 (17)	
Non-pregnant women	25 (35)	31 (43)	16 (22)	
Pregnant women	22 (24)	56 (60)	15 (16)	
Total	91 (31)	152 (51)	53 (18)	

*P values for differences in genotype frequencies between groups were derived using the *χ* ^2^ test, except for rare alleles (*IGF1* rs12579108 and *H19* rs217727), where frequencies were compared using Fisher's exact test.

**Table 3:** Predictors of plasma IGF concentrations overall, in men, non-pregnant women and pregnant women. SNP names are shown in the form of gene names, SNP number (alleles). Correlations are partial correlations for each factor in the final model and total correlation for the model. The most common allele is shown first and the ancestral allele is underlined. Predictors of plasma hormone concentrations were derived using the natural log of plasma concentrations as outcomes by stepwise backward linear regression commencing with a model including subject group (for overall model only), age and common allele frequency for each SNP.

**Groups**	**Predictors**	***r***	***P* value**
Plasma *IGF1*			
Overall	Group	−0.390	<0.001
	Age	−0.350	<0.001
	*IGF2* rs680 (G>A)	0.190	0.002
	*IGF2* rs3741204 (A>G)	−0.206	0.001
	*IGF2* rs1004446 (C>T)	0.200	0.001
	Model	0.501	<0.001
Men	Age	−0.439	<0.001
	Model	0.439	<0.001
Non-pregnant women	Age	−0.405	0.004
	*IGF2* rs680 (G>A)	0.257	0.074
	*IGF2* rs3741204 (A>G)	−0.281	0.050
	*IGF2* rs1004446 (C>T)	0.246	0.089
	Model	0.535	0.003
Pregnant women	Age	−0.197	0.068
	*IGF2* rs680 (G>A)	0.206	0.055
	Model	0.289	0.025
Plasma *IGF2*			
Overall	Group	−0.194	0.002
	*IGF2* rs3741204 (A>G)	−0.158	0.010
	*H19* rs217727 (C>T)	−0.103	0.096
	*IGF2* rs1004446 (C>T)	0.153	0.014
	Model	0.257	0.001
Men	No significant predictors		
Non-pregnant women	No significant predictors		
Pregnant women	No significant predictors		
